# Low-Level Respirable Crystalline Silica and Silicosis: Long-Term Follow-Up of Vermont Granite Workers

**DOI:** 10.3390/ijerph21050608

**Published:** 2024-05-10

**Authors:** Pamela M. Vacek, Robert E. Glenn, John E. Parker

**Affiliations:** 1 Medical Biostatistics Unit, University of Vermont Larner College of Medicine, Burlington, VT 05405, USA; 2 Glenn Consulting Group, 2784 Little Creek Road, Seabrook Island, SC 29455, USA; bobglenn67@comcast.net; 3 Pulmonary and Critical Medicine, Robert C. Byrd Health Sciences Center, West Virginia University, Morgantown, WV 26506, USA; jparker@hsc.wvu.edu

**Keywords:** silicosis, crystalline silica, granite, chest radiograph, exposure

## Abstract

The lifetime risk of silicosis associated with low-level occupational exposure to respirable crystalline silica remains unclear because most previous radiographic studies included workers with varying exposure concentrations and durations. This study assessed the prevalence of silicosis after lengthy exposure to respirable crystalline silica at levels ≤ 0.10 mg/m^3^. Vermont granite workers employed any time during 1979–1987 were traced and chest radiographs were obtained for 356 who were alive in 2017 and residing in Vermont. Work history, smoking habits and respiratory symptoms were obtained by interview, and exposure was estimated using a previously developed job-exposure matrix. Associations between radiographic findings, exposure, and respiratory symptoms were assessed by ANOVA, chi-square tests and binary regression. Fourteen workers (3.9%) had radiographic evidence of silicosis, and all had been employed ≥30 years. They were more likely to have been stone cutters or carvers and their average exposure concentrations and cumulative exposures to respirable crystalline silica were significantly higher than workers with similar durations of employment and no classifiable parenchymal abnormalities. This provides direct evidence that workers with long-term exposure to low-level respirable crystalline silica (≤0.10 mg/m^3^) are at risk of developing silicosis.

## 1. Introduction

The Vermont granite industry has been studied extensively since the 1920s, when the U.S. Public Health Service conducted a comprehensive dust and clinical study that documented a very high prevalence of respiratory morbidity and mortality among workers [1]. This led the Vermont Department of Health’s Division of Industrial Hygiene to implement a program of dust controls in 1938, and to initiate periodic exposure assessment and medical surveillance to monitor its effectiveness. By 1939, all granite manufacturing sheds had installed local exhaust ventilation for all dust-generating sources and by 1949, all granite quarries had switched from dry to wet drilling [2]. The information acquired by this surveillance of Vermont granite workers played an important role in the development of standards for occupational exposure to respirable crystalline silica in the USA [3,4].

In a comprehensive analysis of 5204 historical exposure measurements made in the Vermont granite industry between 1924 and 2004, exposures to respirable crystalline silica were estimated for 22 job categories. Estimates for the categories ranged from 0.03 to 1.07 mg/m^3^ prior to 1940, 0.01 to 0.56 mg/m^3^ during 1940–1949, and 0.01 to 0.10 mg/m^3^ from 1950 [5]. A number of studies confirmed that the reductions in exposure were accompanied by substantial decreases in the prevalence of silicosis. Radiographic surveillance of the workforce found that the prevalence had dropped from 45% in 1937–1938 to 11.3% in 1963 [2,6]; 5.7% of radiographs taken in 1970–1971 had abnormalities considered diagnostic for silicosis [7]. The Vermont Department of Health ended its medical surveillance in 1976, but a radiographic study of 972 Vermont granite workers conducted in 1983 found that seven (0.7%) had silicosis [8]. These previous radiographic studies of the Vermont granite industry included workers who began employment prior to the full implementation of dust controls and thus potentially had some exposure at high levels, as well as workers who had insufficient duration of employment or follow-up for the development of silicosis. 

Workers in epidemiologic studies from other industries with exposure to respirable crystalline silica also had varying durations and concentrations of exposure. Exposure–response analyses from these studies have been used to predict silicosis risk for long-term exposure at specific concentrations, and the estimates have varied widely [9]. The discrepancies are particularly large for low concentrations because very few of the long-term workers in the studies were only exposed to low levels. The risk estimates are highly influenced by workers’ exposure patterns and the models used to quantify exposure–response. The lifetime risk of silicosis for workers with many years of exposure to lower levels of respirable crystalline silica therefore remains unclear. 

The current study is a long-term follow-up of a cohort of Vermont granite workers who were working in the industry at some time during 1979–1987, and most of their exposure levels ranged from 0.01–0.10 mg/m^3^ of respirable crystalline silica. Some of the workers participated in the 1983 radiographic study and their prior radiographic findings were available to assess the progression of any classifiable lung abnormalities found at that time. The goal of this study was to obtain direct evidence about the lifetime risk of silicosis among workers with lengthy exposure to respirable crystalline silica at levels ≤ 0.10 mg/m^3^. 

## 2. Materials and Methods

### 2.1. Study Population

**Figure 1:**
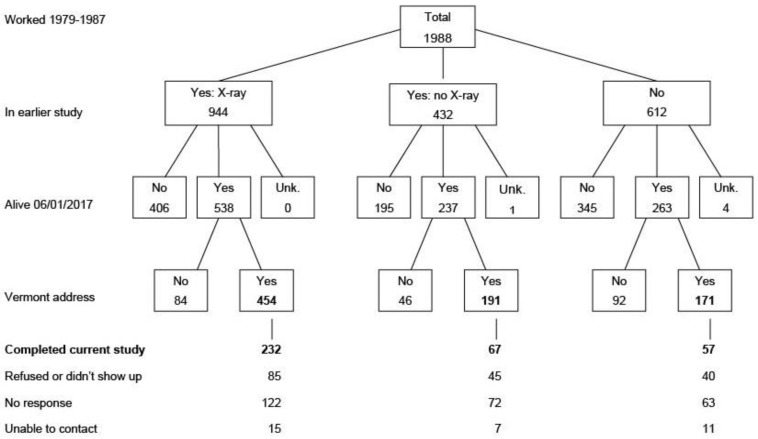
Population employed in the Vermont granite industry during 1979–1987 and flow chart of participant recruitment. Bold numbers indicate eligible workers and recruited participants.

Data from previous studies, pension records and insurance records were used to identify a cohort of 1988 workers who began work in the Vermont granite industry after 1940 and were working at any time between 1979–1987. Radiographic and clinical studies of the workers were conducted during those years; nearly 70% (1376) of the cohort had participated, with 944 having chest radiographs obtained during1983. A private research service was used to ascertain the mortality status of the cohort members and the addresses of those still alive. More than half (1043) of the 1988 workers were still alive as of 6 January 2017 and 816 (78.2% of those still alive) were either permanent or part-time residents of Vermont at that time. Multiple mailings and telephone calls were made to contact the Vermont residents and invite them to participate in the current radiographic study. Of the 816, 290 (34.5%) either could not be reached or did not respond to our letters and calls, and 130 refused to participate. Forty of the 396 who agreed to participate did not keep their appointments, leaving 356 (43.6%) eligible participants who completed the study (Figure 1). The most frequent reasons for refusal to participate were brief employment in the granite industry and having jobs with little or no perceived exposure to granite dust.

### 2.2. Data Collection

A study site was established at the Vermont Granite Museum in Barre and participants scheduled a time to attend during 5 June–17 June 2017. After providing written consent, each participant was interviewed to obtain a detailed work history, including place of employment, job description, and start and end dates for each job held in the granite industry. Information about smoking habits, current respiratory symptoms, and employment in other industries involving potential exposure to respirable crystalline silica was also obtained by interview. Digital chest radiographs were taken from a mobile medical coach with conventional chest radiograph equipment using techniques to optimize images for the detection of subtle changes consistent with pneumoconiosis. Radiographs were reviewed onsite and repeated if film quality was inadequate for the International Labor Office (ILO) classification of pneumoconiosis. 

### 2.3. Silicosis Identification

All radiographs taken for the current study were read by an NIOSH certified B-reader (JEP) who is an expert on surveillance and screening for occupational respiratory diseases. Radiographic findings were classified using the *Guidelines for the Use of the ILO International Classification of Radiographs for Pneumoconioses* and recorded on ILO forms [10]. Findings were considered positive if there were any parenchymal abnormalities consistent with pneumoconiosis and were classified as silicosis if the reader identified small opacities with a profusion of 1/0 or higher and a predominantly rounded shape occurring in any of the six lung zones or identified any large opacity of size A, B or C. Radiographic results from 1983 were used identify participants with evidence of silicosis at that time, as well as silicosis cases among non-participants. The 1983 study used the same criteria as the current study to identify silicosis, but three B Readers independently read each radiograph and silicosis was considered to be present if the findings were classified as silicosis by at least two readers. Evidence of silicosis among deceased cohort members was obtained using cause of death information from a previous mortality study for cohort members who died before 2005 [11] and death certificate information provided by the Vermont Department of Health for deaths occurring during 2005–2016.

### 2.4. Exposure Assessment

Work history information was used to determine the dates of first and last employment in the Vermont granite industry for each study participant and total exposure duration, including any gaps in employment, was calculated as the difference between the dates. Duration of employment for each of the jobs a participant held was similarly calculated and summed over all jobs to determine net exposure duration. Each job was classified into one of 22 categories and assigned the corresponding respirable crystalline silica concentration estimated from a previously developed job-exposure matrix for the Vermont granite industry, whose details have been described in an earlier publication [5]. Briefly, jobs were classified into categories reflecting their exposure potential and the yearly concentration of respirable free silica for each category was estimated for different time periods based on historical exposure measurements, previous studies, information about industrial processes and dust controls, and interviews with long-term employees. Cumulative exposure was computed by multiplying each job’s duration by its estimated exposure concentration and summing over all jobs. Cumulative exposure was divided by net exposure duration to obtain a worker’s average exposure concentration while employed in the granite industry.

### 2.5. Statistical Analysis

Participants were divided into three groups based on their ILO findings (no parenchymal abnormalities consistent with pneumoconiosis, classifiable parenchymal abnormalities not meeting the criteria for silicosis, or silicosis). All workers with silicosis had been exposed to respirable crystalline silica for more than 30 years and they were compared to workers in the other two groups who also had ≥30 years of exposure. Differences between the groups with regard to age at the time of the study and exposure variables (years of first and last exposure, net and total exposure duration, average concentration of exposure to respirable crystalline silica and cumulative exposure) were assessed by analysis of variance, using the Dunnett procedure to adjust for multiple pairwise comparisons. Chi-square tests were used to compare the percentage of workers in each group that had held specific types of jobs, and binary regression was used to compare the prevalence of respiratory symptoms. Differences between groups were considered statistically significant at *p* < 0.05.

## 3. Results

### 3.1. Silicosis among Non-Participants

Among the 945 cohort members who had died prior to the current study, 7 workers had evidence of silicosis indicated on their death certificates and an additional 6 were classified as having silicosis based on radiographs taken during 1983 [8]. The prior radiographic study also identified 3 cases of silicosis among the 683 workers who were alive at the time of the current study but did not participate.

### 3.2. Radiologic Findings

Of the 356 workers who completed the study, 14 (3.9%) had radiographs with evidence of silicosis (the presence of small, rounded opacities with a profusion of 1/0 or higher, or the presence of large opacities). Seven of the workers with silicosis had category 2 profusion scores (two with 2/3 and five with 2/2). One individual with a 2/3 profusion score and one with a 2/2 profusion score had size B large opacities. The remaining seven workers had category 1 profusion scores (three with 1/2, three with 1/1, and one with 1/0). One individual with a 1/2 profusion score and one with a 1/1 profusion score had size A large opacities. Ten of the silicosis cases had radiographic data from the 1983 study and one of them was classified as having silicosis with a profusion score of 1/1 at that time by two of the readers (Table 1). His profusion score had increased to 2/2 by 2017. Another of the silicosis cases was classified as having silicosis by one of the readers in the 1983 study. He had a profusion score of 1/1 at both time points, but large opacities were only observed in 2017.

Eight participants in the current study had parenchymal abnormalities that were consistent with pneumoconiosis but did not meet the criteria for silicosis. All these workers had small opacity profusion scores of 0/1, with half showing a predominantly rounded shape (Table 1). Seven of them had radiographic data from the 1982–1983 study and none had findings consistent with pneumoconiosis at that time. Of the 334 study participants with no evidence of parenchymal abnormalities in 2017, 232 had radiographic data from the1982–1983 study. Four of these workers were classified as having silicosis at that time by one of the three readers, while 33 had findings consistent with pneumoconiosis that did not meet the criteria for silicosis (24 by one reader and 9 by more than one reader). 

### 3.3. Duration of Exposure to Respirable Crystalline Silica

All workers with classifiable parenchymal abnormalities worked in the Vermont granite industry over a period of at least 30 years. Total durations of exposure to respirable crystalline silica, including gaps in employment, ranged from 31 to 51 years for the 14 workers with evidence of silicosis, seven of whom were exposed for 40 or more years. Seven of the eight workers with abnormalities not classified as silicosis had total exposure durations ranging from 30 to 63 years, while one had been part-owner of a granite company for 13 years and said he spent no time on the worksite. Among 334 workers with no classifiable parenchymal abnormalities, total exposure durations ranged from 0.5 to 63 years, but 240 (71.9%) of the workers had been employed in the Vermont granite over a period of 30 or more years.

### 3.4. Exposure Characteristics of Workers Employed ≥30 Years by ILO Classification

Exposure data for workers with parenchymal abnormalities were compared to the 240 workers without abnormalities who had total exposure durations of ≥30 years (Table 2). On average, the workers with no parenchymal abnormalities were significantly younger than either the workers with silicosis or those with classifiable abnormalities that did not meet the criteria for silicosis. Their dates for first and last employment in the industry also were significantly later than those for the two groups of workers with classifiable abnormalities. In addition, 80 (33.3%) were still employed at the time of the study, while all the workers with classifiable abnormalities were retired. Total and net exposure durations were very similar for most workers, indicating that most gaps in employment were short. As expected, neither differed significantly between the workers with and without classifiable abnormalities because all had been employed for at least 30 years. However, average respirable crystalline silica exposure concentrations were significantly lower in workers without abnormalities (mean = 0.05 mg/m^3^, SD = 0.02) than those with silicosis (mean = 0.7 mg/m^3^, SD = 0.01) and those with abnormalities not classified as silicosis (mean = 0.7 mg/m^3^, SD = 0.004), and their average cumulative exposures were correspondingly lower.

Although not significantly different from those with silicosis, the mean age, total exposure duration, net exposure duration, and cumulative exposure were higher in the workers with abnormalities that did not meet the criteria for silicosis. They also tended to have begun and ended work earlier than the workers with silicosis (Table 2).

Forty-two (17.5%) of the 240 workers with exposure durations ≥ 30 years and no parenchymal abnormalities spent all or part of their employment working in the Vermont granite quarries, where respirable crystalline silica levels and silicosis prevalence have historically been lower than in the granite sheds. In contrast, 1 (4.8%) of the 21 study participants with classifiable parenchymal abnormalities worked only in quarries while employed in the Vermont industry and all of the others worked exclusively in the granite sheds, although the difference from workers without abnormalities was not statistically significant. However, the percentage of workers who had held specific types of jobs did differ significantly between workers with and without parenchymal abnormalities (Table 3).

In particular, 2 (14.3%) of the 14 workers with silicosis and 2 (28.6%) of the 7 workers with abnormalities not classified as silicosis had been employed as carvers at some time during their tenure in the granite industry, compared to only 6 (2.5%) of the 240 workers without parenchymal abnormalities. Eight (57.1%) of the workers with silicosis had been employed as cutters, compared to 21.8% of the workers without parenchymal abnormalities. The percentage of workers with abnormalities not classified as silicosis who had worked as cutters (28.6%) did not differ significantly from either the workers with silicosis or those without abnormalities. The percentages of workers who had held other jobs with increased respirable crystalline silica levels, such as polishers and sandblasters, did not differ significantly between the three groups.

### 3.5. Cigarette Use

The smoking habits of the workers with silicosis were similar to those of the workers with exposure durations ≥ 30 years and no classifiable parenchymal abnormalities. One (7.1%) of the workers with silicosis reported that he was currently smoking cigarettes, compared to 23 (9.7%) of the workers without parenchymal abnormalities, but 10 (71.4%) of the workers with silicosis and 144 (61.8%) of the workers with no abnormalities had formerly smoked. None of the 7 workers with parenchymal abnormalities not classified as silicosis was currently smoking and only 3 (42.9%) had smoked in the past, but the percentages were not significantly lower than either of the other groups.

### 3.6. Respiratory Symptoms

The study participants with silicosis tended to report more respiratory symptoms but only the prevalence of dyspnea differed significantly from the participants with lengthy exposure and no classifiable abnormalities (Table 4). Eleven (78.6%) of the workers with silicosis reported shortness of breath while hurrying or walking up a slight hill compared to 41.4% of the workers with no abnormalities. Similarly, 35.7% of the workers with silicosis had shortness of breath while walking at their own pace on level ground, while only 9.3% of those with no classifiable parenchymal abnormalities reported having this symptom. Workers with abnormalities not classified as silicosis had a significantly higher prevalence of dyspnea while hurrying or walking up a slight hill than workers with no abnormalities. In addition, significantly higher proportions reported bringing up phlegm first thing in the morning and having had a chest illness in the last three years that lasted a week.

Smoking status was significantly related to the prevalence of cough, phlegm production, shortness of breath and wheezing. Workers who had never smoked reported the lowest prevalence for all symptoms and current smokers had the highest prevalence for most symptoms. Compared to workers with ≥30 years of exposure and no classifiable parenchymal abnormalities, adjustment for smoking status had little effect on the relative risk of respiratory symptoms for workers with silicosis, but it slightly increased the relative risk of cough and phlegm production for the workers with parenchymal abnormalities not classified as silicosis (Table 4).

## 4. Discussion

Although the dust controls implemented during the 20th century greatly reduced the burden of silicosis among Vermont granite workers, this study clearly demonstrates that the disease has not been eradicated. Among the entire cohort of 1988 workers, a total of 30 silicosis cases were identified: 14 among the 356 study participants and 16 among the 1632 cohort members who had died, were untraceable, or refused to participate in the current radiographic study. This corresponds to an overall silicosis prevalence of 1.5% and represents a minimum because of probable undetected cases among the deceased workers and other non-participants.

The silicosis prevalence of 3.9% among the study participants was somewhat lower than that reported in a previous study that examined surveillance and clinical radiographs from Vermont granite workers who had retired by 1996 [12]. In that study, silicosis was found in 20 (5.7%) of the 350 workers who had been hired after 1940, some of whom were still working at the time of their radiographs. Of the 269 workers whose radiographs had been taken after retirement, 19 (7.1%) had silicosis. In the current study, all workers with silicosis were retired but 82 of the other participants were still employed, corresponding to a silicosis prevalence of 5.1% among retired participants. The lower prevalence in the current study does not appear to be due to duration of exposure or length of follow-up because the participants were exposed for an average of eight years longer and were about eight years older at the time their radiographs were taken than their counterparts in the earlier study. One possible reason for the difference in prevalence is that the earlier study only included retirees with existing radiographs, and these may have been more available for workers who had respiratory symptoms.

A comprehensive analysis conducted by the U.S. Occupational Safety and Health Administration (OSHA) used exposure–response data from a wide range of studies and predicted the silicosis risk associated with 45 years of exposure to 0.1 mg/m3 of respirable crystalline silica [9]. Among radiographic studies with post-employment follow-up, the risk estimates ranged from 6% among Chinese pottery workers [13] to 77% among South African gold miners [14]. An alternative exposure–response modeling of the data from the Chinese pottery workers included changes in exposure patterns over the time yielded, as well as age, sex and smoking [15]. It predicted the silicosis risk at age 65 after 45 years of exposure to be 0.15% over a baseline risk of 1.4% in unexposed workers. The low response rate in the current study precludes an accurate estimation of risk, but the silicosis prevalence of 3.9% that we observed among participants has the advantage of being a direct observation in a group of workers who were only exposed to respirable crystalline silica at levels ≤ 0.1 mg/m3, rather than a prediction based on data from workers with varying levels of exposure.

All 14 of the silicosis cases detected in the current study occurred in workers who had been exposed over a period of more than 30 years. Without routine radiologic surveillance it is impossible to know when the earliest evidence of silicosis appeared in these workers. Two of 10 cases who had radiographs from the 1983 study were classified as having silicosis at that time by at least one of the three readers, but one of them had already been exposed for 27 years and the other for 23 years. This finding, as well the absence of parenchymal abnormalities among participants in the current study who had been exposed for less than 30 years, confirms that, at low levels of respirable crystalline silica, the lung changes indicative of silicosis usually occur after lengthy exposure. 

Among the study participants with exposure durations spanning at least 30 years, average concentrations and cumulative exposures to respirable crystalline silica were higher for the workers who developed silicosis than for those with no classifiable parenchymal abnormalities. This reflects the higher percentage of workers who worked as stone cutters and carvers, and is consistent with a Spanish study of the occupations associated with diffuse interstitial lung disease that found “stone cutting-carving” to be the most frequent activities associated with silicosis [16]. These jobs typically have a greater potential for exposure to respirable crystalline silica than other jobs in the granite industry and represent an area where better dust control and/or adherence to protective measures may be needed.

Radiographs from seven study participants with ≥30 years of exposure showed small opacities consistent with pneumoconiosis, but their profusion scores did not meet the criteria for silicosis. With the small number of workers in this group, there was little statistical power to detect differences from the workers with silicosis, but it is interesting that they tended to be older, had begun working at an earlier date and all had cumulative exposures >2.0 mg/m^3^-years. In contrast, three of the workers with silicosis had cumulative exposures below 2.0 mg/m^3^-years. One of them had a gap in employment, during which he worked in an industry with potentially higher levels of exposure to respirable crystalline silica; thus his cumulative exposure may have been considerably higher than that indicated by his work in the Vermont granite industry. He also had a history of cigarette smoking, as did another of the workers with cumulative exposure below 2.0 mg/m^3^-years. The third worker with lower cumulative exposure who developed silicosis only worked in quarries and never smoked. It is difficult to speculate why the seven workers with low-profusion small opacities indicative of pneumoconiosis did not progress to silicosis. However, four of them had never smoked cigarettes, which accounted for the lower prevalence of some respiratory symptoms and may have reduced the impact of exposure to respirable crystalline silica.

This study highlights the difficulties in conducting long-term follow-ups of occupational cohorts needed to fully understand the lifetime health risks associated with a specific type of industry. Although the Vermont granite industry has a stable workforce and many workers remain in Vermont after retirement, almost half of the cohort working in 1979–1988 had died by 2017. Seven of the deceased had silicosis noted on their death certificates but it is likely that others had undetected lung abnormalities indicative of silicosis. Nearly 80% of those still alive were living in Vermont, but more than half of them either did not respond to numerous contact attempts or refused to participate in the study. Hence, the participants may not be representative of all cohort members who were alive in 2017, particularly because they were significantly older than the non-participants.

Another potential limitation to the study is that the job-exposure matrix used to estimate workers’ exposures was based on exposure measurements made between 1924 and 2004, and it assumes that job-specific concentrations of respirable crystalline silica did not change after 1950 [5]. The majority of measurements were made between 1950 and 1988, and there was no evidence of changes during that time, but it is possible that job-specific reductions in exposure occurred after 1988. Exposures to respirable crystalline silica may therefore have been overestimated for some workers, particularly those with no classifiable abnormalities because their average year of first employment was 1970, 10 years later than the workers with silicosis. On the other hand, some workers’ exposures may have been underestimated because they occasionally did additional part-time or freelance work while employed or after retirement but did not provide enough detail to include these sources in their estimated exposure.

## 5. Conclusions 

This study provides direct evidence that granite workers with long-term exposure to ≤0.10 mg/m^3^ respirable crystalline silica are at risk of developing silicosis, particularly if they are employed as stone cutters and carvers. Although many of the detected cases had simple silicosis with a profusion category of 1 or 2, corresponding to low or moderate radiographic severity, they had an increased prevalence of dyspnea compared to workers with similar smoking histories and no classifiable parenchymal abnormalities. These results reinforce the importance of regulating and monitoring occupational exposure to respirable crystalline silica and justify the periodic radiographic screening of all exposed employees to identify early parenchymal changes indicative of silicosis.

## Figures and Tables

**Table 1:** Summary of radiographic findings for parenchymal abnormalities consistent with pneumoconiosis in 2017.

	Small Opacities				
	Shape 1	Shape 2	Profusion	Large Opacities	Findings from 1983 Study
Silicosis					
	q	t	2/3	0	Negative
	r	q	1/1	A	Silicosis—1 reader
	q	q	1/1	0	Negative
	q	q	1/2	0	Negative
	q	q	2/2	0	Silicosis—2 readers
	q	q	1/1	0	Negative
	q	q	2/2	0	Negative
	q	t	2/2	0	Negative
	q	r	2/3	B	Negative
	q	q	1/0	0	Negative
	q	t	1/2	0	No X-ray
	q	t	1/2	A	No X-ray
	q	t	2/2	0	No X-ray
	q	q	2/2	B	No X-ray
Abnormalities not meeting criteria for silicosis.					
	t	t	0/1	0	Negative
	t	t	0/1	0	Negative
	r	r	0/1	0	Negative
	t	t	0/1	0	Negative
	t	t	0/1	0	Negative
	q	q	0/1	0	Negative
	r	r	0/1	0	Negative
	q	q	0/1	0	No X-ray

**Table 2:** Characteristics of granite workers exposed to respirable crystalline silica for ≥30 years by ILO classification.

				Classifiable Parenchymal Abnormalities					
	No Classifiable Abnormalities (N = 240)			Not Classified as Silicosis (N = 7)			Silicosis (N = 14)		
	Mean	SD	Range	Mean	SD	Range	Mean	SD	Range
Age in 2017	69	10.1	50–99	84 *	7.6	72–94	79 *	6.8	60–87
Year began work	1970	9.9	1944–1987	1954 *	8.6	1943–1966	1960 *	7.0	1952–1978
Year ended work	2009	8.4	1984–2017	1999 *	8.4	1989–2009	2001 *	8.7	1990–2016
Total exposure duration (years)	39.3	6.2	30–63	44.6	10.1	30–63	40.7	5.8	31–51
Net exposure duration (years)	38.2	6.5	16.0–63.5	43.3	10.9	30–63	38.5	7.9	25–51
Average concentration (mg/m^3^)	0.05	0.02	0.01–0.07	0.07 *	0.00	0.07–0.08	0.07 *	0.01	0.02–0.07
Cumulative exposure (mg/m^3^ years)	1.92	0.98	0.27–4.44	3.06 *	0.95	2.10–5.01	2.55 *	0.81	0.57–3.57

* Significantly different from workers with no classifiable abnormalities (*p* < 0.05).

**Table 3:** Granite industry jobs held by participants employed ≥30 years.

		No Abnormalities		Classifiable Parenchymal Abnormalities			
				Not Classified Silicosis		Silicosis	
Job Category	Exposure (mg/m^3^)	N	%	N	%	N	%
Boxer	0.04	22	9.2	0	0.0	0	0.0
Carver	0.07	6	2.5	2	28.6 *	2	14.3 *
Crane operator	0.05	20	8.3	0	0.0	0	0.0
Cutter	0.07	52	21.7	2	28.6	8	57.1 *
Foreman	0.05	10	4.2	0	0.0	0	0.0
Grinder	0.07	4	1.7	0	0.0	0	0.0
Laborer	0.10	10	4.2	0	0.0	0	0.0
Lumper	0.06	34	14.2	0	0.0	1	7.1
Maintenance worker	0.07	23	9.6	0	0.0	0	0.0
Polisher	0.07	48	20.0	2	28.6	4	28.6
Sandblaster	0.07	52	21.7	2	28.6	5	35.7
Sawyer	0.06	40	17.2	1	14.3	3	21.4

* Significantly higher than the percent of workers with no classifiable abnormalities (*p* < 0.05).

**Table 4:** Prevalence of respiratory symptoms compared to subjects with no parenchymal abnormalities in participants employed ≥30 years. Relative risk (RR) and confidence interval (CI) unadjusted and adjusted for smoking status.

	No Abnormalities	Abnormalities Not Classified Silicosis			Silicosis		
	N (%)	N (%)	RR (95% CI)	Adj. RR (95% CI)	N (%)	RR (95% CI)	Adj. RR (95% CI)
Cough first thing in morning in winter	0 (23.9%)	2 (28.6%)	1.19 (0.36–3.93)	1.44 (0.43–4.78)	6 (42.9%)	1.79 (0.94–3.41)	1.41 (0.80–2.51)
Cough during day or night in winter	46 (19.3%)	2 (28.6%)	1.48 (0.45–4.93)	1.73 (0.52–5.72)	5 (35.7%)	1.86 (0.88–3.93)	1.78 (0.85–3.74)
Cough for ≥3 month of the year	55 (23.1%)	2 (28.6%)	1.24 (0.37–4.08)	1.54 (0.47–5.07)	5 (35.7%)	1.55 (0.74–3.24)	1.31 (0.67–2.58)
Bring up phlegm first thing in morning in winter	64 (27.0%)	4 (57.1%)	2.12 * (1.08–4.16)	2.62 * (1.43–4.80)	5 (35.7%)	1.32 (0.64–2.75)	1.40 (0.67–2.91)
Bring up phlegm during day or night in winter	41 (17.2%)	2 (28.6%)	1.66 (0.50–5.53)	2.20 (0.69–6.97)	5 (35.7%)	2.07 (0.97–4.42)	2.10 (0.98–4.49)
Bring up phlegm for ≥3 month of the year	52 (22.4%)	2 (28.6%)	1.27 (0.39–4.21)	1.69 (0.53–5.42)	4 (28.6%)	1.27 (0.54–3.02)	1.35 (0.57–3.20)
Increased cough/phlegm lasting ≥3 weeks in past 2 years	40 (16.7%)	1 (14.3%)	0.85 (0.14–5.36)	0.90 (0.14–5.66)	4 (28.6%)	1.71 (0.71–4.10)	1.71 (0.71–4.14)
Short of breath hurrying or walking up slight hill	98 (41.4%)	5 (71.4%)	1.73 * (1.06–2.83)	1.63 * (1.05–2.53)	11 (78.6%)	1.90 * (1.39–2.60)	1.68 * (1.24–2.27)
Short of breath walking level ground at own pace	22 (9.3%)	1 (14.3%)	1.54 (0.24–9.86)	1.52 (0.24–9.70)	5 (35.7%)	3.85 * (1.72–8.63)	3.46 * (1.53–7.79)
Wheezing attack in last 12 months	54 (22.6%)	1 (14.3%)	0.63 (0.10–3.94)	0.81 (0.13–5.00)	6 (42.9%)	1.90 (0.99–3.63)	1.64 (0.86–3.10)
Ever had wheezing attack with shortness of breath	44 (18.7%)	0 (0.0%)	0.00 (0.00–)	0.00 (0.00–)	5 (35.7%)	1.91 (0.90–4.04)	1.87 (0.89–3.93)
Woken at night with shortness of breath in last 12 months	20 (8.5%)	1 (14.3%)	1.68 (0.26–10.81)	1.56 (0.24–10.06)	0 (0.0%)	0.00 (0.00–)	0.00 (0.00–)
Chest illness within 3 years that lasted a week	30 (13.4%)	3 (42.9%)	3.20 * (1.28–8.01)	3.14 * (1.21–8.14)	2 (16.7%)	1.24 (0.34–4.60)	1.19 (0.32–4.41)

Sample size for subjects with no abnormalities ranged from 224 to 239 due to missing data. Adjusted relative risk includes adjustment for smoking status. Upper confidence limits not estimable for zero prevalence. * Significantly higher than workers with no classifiable abnormalities (*p* < 0.05).

## Data Availability

The data collected for this study cannot be shared publicly to protect the privacy of the participants, who were assured of confidentiality prior to providing consent.
